# Patients’ perspectives of care for type 2 diabetes in Bangladesh –a qualitative study

**DOI:** 10.1186/1471-2458-14-737

**Published:** 2014-07-21

**Authors:** Christopher P Lewis, James N Newell

**Affiliations:** 1 Airedale General Hosptial, Steeton BD20 6TD, UK; 2 Nuffield Centre for International Health and Development, University of Leeds, Leeds, UK

## Abstract

**Background:**

Worldwide, type 2 diabetes affects approximately 220 million people and is the cause of 1.1 million deaths each year, 80% of which occur in low and middle income countries (LMICs). Over the next 20 years, prevalence is expected to double worldwide and increase by 150% in LMICs. There is now a move towards improving care for diabetes. However no information on patients’ needs, perceptions and experiences is available, hindering effective and appropriate changes in policy and practice. We developed a study with the objective of understanding patients’ experiences of treatment for type 2 diabetes.

**Methods:**

During January 2011, we conducted in-depth interviews in five sites across two administrative districts of Bangladesh, purposefully chosen to represent different geographic regions and local demographics In total, we conducted 23 (14 male, 9 female) individual interviews across the 5 sites, to gain insight into patients’ understanding of their diabetes and its management.

**Results:**

Patients’ levels of knowledge and understanding about diabetes and its management is depended on where they received their initial diagnosis and care. Away from specialist centres, patients had poor understanding of the essential of diabetes and its management. No appropriate written or verbal information was available for a significant number of patients, compounded limited knowledge and understanding of diabetes by healthcare professionals. Patients felt that with improved provision of appropriate information they would be able to better understand their diabetes and improve their role in its management. Access to appropriate diagnosis and subsequent treatment was restricted by availability and costs of services.

**Conclusion:**

Effective, appropriate and essential healthcare services for diabetes in Bangladesh is extremely limited, a majority of patients receive suboptimal care. Site of diagnosis will impact significantly on the quality of information provided and the quality of subsequent treatments. Although appropriate services are available at some specialist centres, the inability of patients to pay for routine tests and check-ups prevents them from receiving timely diagnoses and appropriate continuity of care. The double burden of communicable diseases and diseases is now a well-recognised. Emphasis must be placed on developing appropriate and effective preventive strategies to address this burden.

## Background

Worldwide, type 2 diabetes affects approximately 220 million people [1] and is the cause of 1.1 million deaths each year, 80% of which occur in low and middle income countries (LMICs) [2]. Over the next 20 years, prevalence is expected to double worldwide [1] and increase by 150% in LMICs [1-3]. In SE Asia, although epidemiological data is lacking and estimates as to the burden of diabetes across the region vary, prevalence (Tables 1 and 2) has risen more rapidly than in any other region: India now has an estimated 32.7 million affected people, and this number is expected to double by 2025. A similar situation exists in other SE Asian countries [3,4]. The rapid rise in the prevalence of non-communicable diseases such as cardiovascular disease and diabetes means that non communicable, chronic diseases are predicted to contribute to 80% of the burden of disease and cause 7 out of every 10 deaths in LMICs by 2020 [3,5,6]. In these countries diabetes predominantly affects individuals between the ages of 35 and 65, in contrast to higher income countries where prevalence is highest in the 65+ age group [3]. Globally, and particularly within SE Asia, diabetes represents a major public health challenge, and a significant obstacle to economic growth and development [7].

**Table 1:** **The estimated prevalence diabetes mellitus in South East Asia 2010 **[3,4]

**Country/territory**	**Population (20–79) (000 s)**	**Total number of diabetes cases (000 s)**	**Prevalence of diabetes (%) in the 20–79 age-group 2010**				
			**National**	**Rural**	**Urban**	**Male**	**Female**
Bangladesh	93 862	5 680	6.1%	4.4	11.0	5.4	6.1
Bhutan	413	12	2.9%	2.4	8.1	2.6	2.5
India	713 498	50 767	7.1%	5.6	11.2	6.8	5.8
Maldives	186	12	6.5%	3.1	14.5	3.1	5.0
Mauritius	877	14	17.0%	11.2	18.9	15.2	14.5
Nepal	15 556	511	3.3%	2.5	6.8	2.5	3.1
Sri Lanka	13 339	1529	11.5%	8.3	23.5	10.6	11.0
**SEA Total**	**837 732**	**58 658**	**7.0%**	**5.4**	**11.3**	**6.6**	**6.6**

**Table 2:** **Predicted diabetes prevalence in South East Asia for 2030 **[3-5]

**Country/territory**	**Population (20–79) (000 s)**	**Total number of diabetes cases (000 s)**	**Prevalence of diabetes (%) in the 20–79 age-group 2030**				
			**National**	**Rural**	**Urban**	**Male**	**Female**
Bangladesh	140 679	10 423	7.4	4.9	11.3	6.4	7.6
Bhutan	589	25	4.3	3.2	8.5	6.3	6.1
India	1 017 413	87 035	8.6	9.1	8.2	7.6	8.5
Maldives	285	26	9.0	3.9	15.2	6.5	6.8
Mauritius	1 035	224	21.7	13.1	3.7	22.9	23.9
Nepal	25 391	1 071	4.2	2.9	7.3	3.4	4.7
Sri Lanka	14 493	2 157	14.9	8.9	28.9	13.5	16.5
**SEA Total**	**1 199 885**	**100 874**	**8.4**	**6.3**	**11.5**	**9.5**	**10.6**

Fundamental components of good diabetes care are good diagnosis, good access to affordable treatments and good patient education and awareness [8,9]. Patient-centred care focuses on each patient as an individual, addressing their specific social, cultural and religious needs to provide a treatment package appropriate for that individual [10]. In SE Asia, the fundamental components are lacking, and understanding of patients’ needs, perceptions and treatment experiences, vital to providing patient-centred care, is currently very limited.

In Bangladesh, government health provision currently focuses on communicable diseases: diabetes care is provided primarily by the Diabetes Association of Bangladesh (BADAS) which provides specialist clinics and tertiary-level specialist hospitals. BADAS also has a research institute, the Bangladesh Institute of Research and Rehabilitation in Diabetes, Endocrine and Metabolic Disorders (BIRDEM), which has a main hospital providing specialist services in Dhaka and a network of specialist diabetes centres across Bangladesh. There is now a political move towards improving care for diabetes (as well as other non-communicable diseases [11,12]). However, no information on patients’ needs, perceptions and experiences is available, hindering effective and appropriate changes in policy and practice. We therefore developed a study with the objective of understanding patients’ experiences of treatment for type 2 diabetes.

## Methods

During January 2011, we conducted in-depth interviews in five sites across two administrative districts of Bangladesh. These sites were purposefully chosen to represent different geographic regions and local demographics (Table 3). Sites A, B & C were all within the recognised Dhaka Metropolitan City limits; sites D & E were in Sylhet division in north-eastern Bangladesh, chosen to represent a major urban centre outside of the capital Dhaka.

**Table 3:** Areas and sites visited

**Site**	**Location**	**Typography**	**No of patient interviews conducted**	**No of Healthcare professionals interviews conducted**	**Interview location**
**A**	Dhaka	Urban	6	2	BIRDEM clinic
**B**	Dhaka	Urban	3	2	Private practice
**C**	Dhaka	Peri –Urban Slum	5	1	Outreach centre
**D**	Sylhet City	Urban	5	1	BADAS Hospital
**E**	Bianibazar	Rural	4	2	Rural Upazilla level Health Clinic

In total, we conducted 23 (14 male, 9 female) individual interviews across the 5 sites, to gain insight into patients’ understanding of their (type 2) diabetes, its root causes and its effective management. The number of patient interviews conducted at each site was based on the willingness of patients attend for diabetes related care to discuss their treatment. We also asked patients to describe their personal experiences during treatment, including any difficulties or positive experiences that they had encountered. Interviews were held at private practitioners’ surgeries (sites B, C), BIRDEM associated clinics (Site A), specialist tertiary level BADAS hospitals (site D) and sub district level healthcare centres (site E) (Table 3). Each of these centres had healthcare practitioners of differing levels of qualification, training and experience in line with the requirements for employment at each centre. We also asked a total of 8 healthcare professionals about their experiences of diabetes service provision (Table 3).

During interviews the interviewer took notes and made audio recordings. Interview transcripts were coded and emergent themes identified. Subsequent interviews were adjusted in the light of emerging themes which arose from initial interviews. All respondents gave written informed consent before each interview. Ethics approval was granted by the Bangladesh Medical Research Council and the University of Leeds. RATS guideline for reporting qualitative studies were adhered at all points during the study.

## Results

### Awareness and understanding of diabetes and its effective management

Patients’ levels of knowledge and understanding about diabetes and its management depended on where they received their initial diagnosis and subsequent care. This was due to variations in the availability of and access to appropriate information, alongside disparities in healthcare worker knowledge and training on diabetes. Patients reported that there choice of clinic was based predominantly on geography, either the nearest possible healthcare centre or in rural cases, the only available healthcare centre. Men’s and women’s responses were similar across all districts. Only those attending the specialist’s clinics had travelled specifically to attend these centres. Patients interviewed at the BIRDEM clinic in Dhaka (Site A) had good awareness and understanding of type 2 diabetes as a result of a comprehensive education programme based on provision of both verbal and written information. These interviewees were aware of the complications of diabetes and the important role that self-care played in minimising the risk of future illness. They reported that they felt this was key in helping them to better manage their diabetes and had helped them to reduce the occurrence of medical complications.

*“Diabetes is the mother of all diseases … it is not easy, but discipline is essential … If I don’t do things right, I can’t go to work and I will become very ill.”* (Patient 1, Site A).

Those patients who received treatment away from BIREDM-associated specialist centres had poor understanding of the essential of diabetes and its management. They had only limited knowledge of how to effectively modify their lifestyle, and felt they had neither the financial means nor the knowledge to obtain and correctly take prescribed medicines. Little or no accessible information in appropriate forms was available for these patients, both when they received their diagnosis and at subsequent consultations.

*“When I eventually found out that I had diabetes … after I could afford to pay for the tests, I was told not to eat too many sweet things and to take some medicines … but how or why I should be taking them was not explained.”* (Patient 3, Site B).

Patients interviewed at the rural upazilla-level clinic (site E) had only very limited awareness of diabetes and its effective management. No appropriate written or verbal information was available for these patients: this problem was compounded by self-reported limited knowledge and understanding of diabetes by healthcare professionals, which proved a significant barrier to provision of appropriate information at the diagnostic and subsequent consultations. Those patients undergoing treatment at private practitioners had some understanding of diabetes, although less than that of patients attending specialist BIRDEM clinics or tertiary level centres. Patients of private practitioners reported that they had been encouraged to change their diet, but not given enough advice or information on to do this in their specific social and economic situations.

*“I got told to take more fruits and vegetables, but they are expensive … I also eat fewer sweet things, but I’m not always sure what is best to eat.”* (Patient 2, Site C).

Patients at all sites felt that with improved provision of appropriate information they would be able to better understand their diabetes and improve their role in its management.

Although patients at all sites reported fasting during the month of Ramadan, only the specialist BIRDEM Clinic (Site A) and the BADAS hospital (Site D) provided advice as to how to modify dietary and medication routines to coincide with periods of fasting. Patients at other sites reported that they felt their health suffered during the fasting period (reporting headaches, weakness and general malaise), but could not specify if they thought this was as a result of their diabetes or other factors.

### Availability and costs of diagnosis and care

In all regions and at all sites, access to appropriate diagnosis and subsequent treatment was restricted by availability and costs of services. Where facilities were available they were placed under huge pressure by a combination of limited resources and the large numbers of patients presenting for care, impacting on their ability to provide comprehensive treatment.

Limited availability of services was most marked in rural and peri-urban slum areas where patients reported that they felt there were no effective medical services available to meet even their most basic needs within easy travelling distance. This fact, compounded by poor community awareness and understanding of diabetes meant that the patients spoken to in these areas had generally received a diagnosis as a result of incidental findings when they sought medical care for other problems such as tuberculosis, or as a result of a visit to a tertiary care centre for treatment of severe complications of poorly controlled diabetes.

*“I only heard about diabetes because one of my husband’s customers in his rickshaw told him about his own illness … then I had to go and find somewhere to get help.”* (Patient 1, Site C).

In peri-urban slum areas, continuity of care in those with a confirmed diagnosis of type 2 diabetes was extremely poor. Patients reported that lack of availability of basic government/NGO medical services meant a local private medical practitioner was the only option; and high costs meant they went only when they felt very ill or to seek care for serious problems associated with poor diabetes control such as foot ulcers and recurrent skin infection. Patients reported that they felt the service given to them by these private practitioners was not comprehensive, as they had received only limited information about how to manage their illness.

In rural areas, patients at community clinics reported that they had to travel to the local sub-district hospital for even the most basic of consultations, while simple blood tests or oral glucose tests required a trip to the nearest BADAS specialist hospital because the sub district hospital lacked the basic facilities to provide diagnostic and follow up care. Patients reported that this caused them significant problems in terms of time and cost, as the specialist hospital was some 70 kilometres away; as a result, most delayed seeking medical care until complications became debilitating.

Only at the BIRDEM clinic in Dhaka were patients routinely offered regular comprehensive checkups including full cardiovascular, renal and eyesight examinations. This service was welcomed by patients attending this specialist clinic as they recognised the importance of these regular and thorough examinations, rather than just blood glucose level monitoring, as essential to appropriate diabetes care.

*“I need to get my eyes and my heart checked when I come, it’s best to make sure I have no problems … I can get it all done here … although it’s hard to afford … but it’s for the best.”* (Patient 4, Site A).

While the BIRDEM clinic was able to provide the most comprehensive care for diabetes, interviews with healthcare professionals there highlighted their concerns, also echoed in subsequent patient interviews, that the service provided by this centre cannot meet the level of demand placed upon it. Healthcare professionals felt that individual patient care was suffering as a result of the huge numbers of patients seeking treatment and the demands this placed on their already limited resources.

*“Patients don’t just come from the local areas, they come from outside Dhaka – they know we provide a good service.”* (Practitioner 1, Site A).

This high demand meant that patients could not be given an approximate appointment time, meaning that patients coming to the centre generally had to allow a whole day for a consultation, which impacted on how often they came for routine appointments because of the time spent, often resulting in the loss of a day’s work and income.

At all sites, access to effective treatment was hindered by lack of essential clinical facilities and adequate training of healthcare workers. This was a situation recognised by all healthcare professionals interviewed, who reported that training on diabetes care could be improved, and, if complemented by provision of better diagnostic and essential medication, would enable them to provide a higher standard of care.

In all regions and at all sites the cost of initial diagnosis, subsequent care and essential medications placed a huge burden on all patients interviewed. These high costs repeatedly led to respondents delaying initial treatment seeking, prevented them from accessing comprehensive and continued healthcare, and severely hindered their ability to buy medications vital for effective management of their illness.

*“I only take medicines when I can afford them; it is too much to pay for them all the time … I have to think of my family.”* (Patient 3, Site C).

At the BIRDEM clinic in Dhaka, although some basic services at first consultation are provided free, patients reported that the relatively high cost of initial diagnostic consultations and subsequent testing had prevented them from receiving a timely diagnosis and thorough medical checkup. This meant that they suffered considerably from side effects of poor glucose control for lengthy periods while they saved and/or borrowed piecemeal from friends and family the funds to be able to afford the initial tests. Healthcare professionals working at the clinic reported helping patients financially so they could receive an initial diagnosis or have continued access to regular essential consultations. The cost of initial diagnostic testing is 500 Taka ($8), two to three days’ average wages [12], well beyond the means of many. Patients reported that they felt their long term health had suffered from having to wait until they could afford diagnosis before they sought care.

*“We all [the healthcare workers at the clinic] sometimes have to go into our own pockets, these patients struggle to afford proper care, and it is our duty to help them if we can.”* (Practitioner 2, site A).

In the peri-urban slum areas visited, all patients reported that they could not access medication or basic diabetic healthcare services primarily because of inhibitory high costs. Patients reported going to local medical practitioners to seek care for the initial symptoms associated with their diabetes. Their decision to visit these practitioners was based on location and cost compared to specialised centres. Patients that visited these practitioners in this area often received contradictory information about effective care for diabetes, and little or no continuity of care.

*“I go to the local place because it is cheapest and I can get there easily. I only go when I feel very bad though … it still costs a lot.”* (Patient 1, Site C).

Patients reported that the high cost of medications meant they took them only when they felt they could justify the cost, usually when they felt severely ill. Two patients from the peri-urban slum areas who had been to a private practitioner had been unable to afford any medications since initial diagnosis. These patients showed clear signs of advanced dermatological and ophthalmic complications of diabetes.

## Discussion

We have added to the evidence showing that provision of effective, appropriate and essential healthcare services for diabetes in Bangladesh is extremely limited and the majority of patients receive suboptimal care. This situation requires immediate and urgent attention, as has recently been called for in both a recent World Bank report and Health literature [13,14]. Poor awareness and understanding of diabetes, its causes, presentation and effective treatment often results in delay of initial treatment–seeking, resulting in long periods of ill health and reduced productivity, impacting significantly on individual patients and their families. Access to care is limited in all regions for both men and women, most markedly in the peri-urban slum and rural areas: this problem is further compounded by high costs of treatment, lack of basic medical and diagnostic equipment and small numbers of appropriately trained healthcare professionals. These factors all act to hinder initial treatment seeking, appropriate patient management and provision of continual and effective care. This is a qualitative study, so we make no claims about generalizability, since choice of sites and sampling methods may have led to findings that are not applicable across Bangladesh. However, the emerging themes are plausible, accord with experience and existing evidence, and provide information that should inform development of policy and capacity-building to meet the growing challenge of diabetes in Bangladesh.

Patients report lack of access to adequate care as being fundamental to the ongoing difficulties they encounter in managing their diabetes. Where services are available in urban centres at limited specialist clinics, they are operating at overcapacity, undermining the provision of effective care to those patients attending for consultation. Only further investment and scale up of these services with increased healthcare professional recruitment will allow for the increased number of patients seeking care to be provided with appropriate levels of initial diagnosis, illness education and subsequent ongoing treatment. To address this chronic lack of service provision, there is a need for, at a minimum, outreach clinics in peri-urban slum and rural areas which raise awareness and understanding of diabetes and encourage people to come forward for screening and diagnosis, and which can refer patients with complicated medical problems for specialist services. Where patients attend private practitioners they often receive contradictory information and are referred to specialised services only once severe complication of diabetes develop. Improving the medical education of these practitioners, allowing them to detect and manage routine cases of diabetes effectively alongside a well established referral system for complicated cases, would help to correct this situation. In rural areas, effective and timely diagnosis and subsequent appropriate treatment is undermined by chronic lack of services, further compounded by limited availability of medical and diagnostic equipment. To address these issues, policy and practice should be developed which seeks to provide basic care for diabetes and other non-communicable diseases alongside the already well-established communicable disease programmes implemented in these regions.

Where a patient receives their diagnosis will impact significantly on the quality of information they are provided with to promote their self care and also the quality of subsequent treatments. The BIRDEM clinic in Dhaka provides a good model of basic care, offering specialist counselling, providing supportive information to newly diagnosed patients and making available appropriate medical services to prevent and manage known complications of diabetes. These services are not available in other areas. This lack of information at these sites prevents patients from becoming involved as active participants in improved self-care. Provision at diagnosis of suitable information in an appropriate written, visual or verbal form providing essential information about diabetes, its causes and treatment would contribute significantly to improving health outcomes.

Although appropriate services are available at some specialist centres, the inability of patients to pay for routine tests and check-ups still prevents them from receiving timely diagnoses and appropriate continuity of care. Patients delay routine appointments and borrow to cover the cost of routine tests. To encourage individuals to come forward for screening, initial diagnosis and regular care, the cost of these consultations should be reduced to a level which is affordable in these communities. In the poorest communities, packages of low cost and comprehensive treatments which offer regular and basic checkups and generic low cost medications at the lowest possible price must be made available. This would contribute to significantly improving the health of diabetics living in poverty in all areas of Bangladesh.

The increasing double burden of communicable diseases and diseases primarily associated with lifestyle such as diabetes is now a well recognised phenomenon in low and middle income countries, including Bangladesh [15-18]. Emphasis must be placed on addressing this burden, by developing appropriate and effective preventive strategies to try to reduce the increasing incidence of diabetes and other non-communicable diseases. Estimates of prevalence of diabetes vary widely and a better understanding of the scale of burden due to diabetes is needed to develop policies and practice which address both prevention and treatment for diabetes in Bangladesh in an appropriate and sustainable manner: robust epidemiological data is needed.

## Conclusion

Effective, appropriate and essential healthcare services for diabetes in Bangladesh is extremely limited, a majority of patients receive suboptimal care. Site of diagnosis will impact significantly on the quality of information provided and the quality of subsequent treatments. Although appropriate services are available at some specialist centres, the inability of patients to pay for routine tests and check-ups prevents them from receiving timely diagnoses and appropriate continuity of care. The double burden of communicable diseases and diseases is now a well-recognised. Emphasis must be placed on developing appropriate and effective preventive strategies to address this burden.

## Competing interests

The authors declare that they have no competing interests.

## Authors’ contributions

CPL was involved in all stages of the study including study design, data collection, analysis and manuscript production. JN contributed to study design and manuscript production. Both authors read and approved the final manuscript.

## Pre-publication history

The pre-publication history for this paper can be accessed here:

http://www.biomedcentral.com/1471-2458/14/737/prepub
